# Inflammation Exacerbates Congenital Zika Virus Infection and Naringenin Provides Protective Effects

**DOI:** 10.3390/v18060615

**Published:** 2026-05-28

**Authors:** Anna Cláudia Calvielli Castelo Branco, Yasmim Álefe Leuzzi Ramos, Carolina Manganeli Polonio, Nagela Ghabdan Zanluqui, Lilian Gomes de Oliveira, Jean Pierre Schatzmann Peron, Fábio Seiti Yamada Yoshikawa, Daniel Pereira Sousa, Laura Luiza Moreira da Silva Dias, Emanuella Sarmento Alho de Sousa, Tamiris Azamor da Costa Barros, Elyzabeth Avvad-Portari, Zilton Farias Meira De Vasconcelos, Amaro Nunes Duarte-Neto, Naiura Vieira Pereira, Mirian Nacagami Sotto, Maria Notomi Sato

**Affiliations:** 1 Laboratory of Medical Investigation in Dermatology and Immunodeficiencies (LIM-56), Department of Dermatology, Faculdade de Medicina, Instiuto de Medicina Tropical de SP, Universidade de São Paulo, São Paulo 05403-000, Brazil; cbranco.anna@gmail.com (A.C.C.C.B.); yasmimleuzzi@gmail.com (Y.Á.L.R.); faseiti@gmail.com (F.S.Y.Y.); danielsousa@usp.br (D.P.S.); lauraluizamsd@usp.br (L.L.M.d.S.D.); emanuellasarmento@usp.br (E.S.A.d.S.); naiurav@gmail.com (N.V.P.); 2 Department of Immunology, Institute of Biomedical Sciences, University of São Paulo, São Paulo 05508-000, Brazil; cmanganelipolonio@bwh.harvard.edu (C.M.P.); nagelaghabdan@gmail.com (N.G.Z.); lilian._gomes@hotmail.com (L.G.d.O.); jeanpierre.schatzmannperon@umassmed.edu (J.P.S.P.); 3 UMass Chan Medical School, Department of Neurology, Worcester, MA 3050, USA; 4 Division of Molecular Immunology, Medical Mycology Research Center, Chiba University, Chiba 260-8673, Japan; 5 Instituto Fernandes Figueira, Fundação Oswaldo Cruz FIOCRUZ, Rio de Janeiro 22250-020, Brazil; tamiris.barros@fiocruz.br (T.A.d.C.B.); bethavvad@gmail.com (E.A.-P.); zilton.vasconcelos@fiocruz.br (Z.F.M.D.V.); 6 Department of Pathology, Medicine School of University of Sao Paulo, Sao Paulo 05403-000, Brazil; amaro.ndneto@hc.fm.usp.br (A.N.D.-N.); mnsotto@usp.br (M.N.S.)

**Keywords:** Zika virus infection, placenta, inflammation, flavonoid naringenin

## Abstract

Zika virus (ZIKV) infection during pregnancy is a critical driver of Congenital Zika Syndrome (CZS), yet the mechanisms of pathogenesis at the placental barrier remain incompletely understood. This article is a translational, observational, and experimental study combining clinical placental analyses, placental explants cultures and in vivo murine model to investigate the mechanisms involved in ZIKV infection. We evaluate the histopathological analyses to verify presence of inflammation in ZIKV-infected human placentas from newborns with CZS and without CZS (N-CZS), identifying more intense Hofbauer cell hyperplasia, villitis and decidual inflammation in CZS group. Moreover, placental immunohistochemistry analyses identified decreased TLR4 expression in the villi and reduced TNF and IL-10 levels across placental layers of CZS group. Next, we investigated the effects of inflammation on viral replication and explored whether the flavonoid Naringenin (NGN) could modulate this inflammation. Using a placental villous explant model, we verified that inflammation induced by LPS exacerbates viral replication and pathological markers. Notably, treatment with the NGN rescued the inflammatory and virological outcomes. These findings were further validated in a murine model of congenital infection, where NGN administration alleviated microcephaly-related structural alterations in ZIKV-exposed neonates. Our results indicate that placental inflammation is a key provocateur of ZIKV replication and subsequent fetal brain malformation. Furthermore, we identify NGN as a promising bifunctional antiviral and anti-inflammatory candidate for mitigating the developmental impacts of ZIKV infection.

## 1. Introduction

Zika virus (ZIKV), a member of the Flaviviridae family, was first isolated in 1947 from rhesus monkeys in the Zika Forest in Uganda [1], with the first human cases reported in Nigeria in 1954. Microcephaly and Guillain-Barré syndrome in adults were identified during the French Polynesia outbreak and in the ZIKV outbreak in the Americas between 2015 and 2017. The World Health Organization declared a PHEIC (Public Health Emergency of International Concern) in February 2016. Although the overall global incidence has declined, recent outbreaks were reported in India, Cambodia, Thailand, and Singapore in 2024, demonstrating its persistent circulation [2]. Despite significant progress in the development of ZIKV vaccine candidates since 2015, no licensed ZIKV vaccines or antiviral therapies are currently available for treatment [2].

ZIKV can be transmitted vertically leading to infection of fetal brain and resulting in primary (congenital) ZIKV syndrome (CZS). CZS encompasses a range of neurological, ophthalmological, audiological and skeletal abnormalities, with microcephaly, the most shocking feature, being characterized by an intense inflammatory imbalance that correlates with disease severity [3]. In a meta-analysis of 1548 pregnant women across 13 studies, one third of live-born children with prenatal ZIKV exposure had at least one anomaly compatible with CZS [4]. Although children born with CZS have a significantly higher risk of cause-specific mortality [5], just the exposure to ZIKV in utero, even without CZS development, is already linked to a higher risk of neurodevelopmental delay in early childhood, with the timing of maternal infection being a significant predictive risk factor [6].

It is well acknowledged that ZIKV infection during pregnancy leads to severe placental injury [7], associated with hyperplasia of placental Hofbauer cells in the chorionic villi and numerous histiocyte-like cells in the decidua [8]. However, these histological changes cannot be predictive of the impact on fetal outcome [7]. Placenta of ZIKV-infected mothers displays excessive inflammation and dysfunctional vascular permeability, with upregulation of multiple markers, such as matrix metalloproteinases, IFN-γ, TNF-α, RANTES/CCL5, VEGFR-2 and brain-derived neurotrophic factor (BDNF), the latter being important for brain development and predictive of the fetal brain changes [7]. Nevertheless, a deeper understanding of the role of placental cells in facilitating ZIKV vertical transmission and fetal neuropathogenesis is not fully achieved. Moreover, whether acute or chronic placental inflammation may favor a viral infection needs clarification. As pregnant women are prone to urinary tract infections, ascending infections may occur, increasing the risk of maternal and neonatal morbidity and mortality [9,10]. In addition, periodontal diseases caused by multiple Gram-negative bacteria affects pregnancy outcomes, showing that the gingival biofilm may directly affect the fetal–placental unit subsequent to bacteremia. Inflammatory mediators secreted by the subgingival inflammatory site are carried to the fetus-placental unit, where they then cause an inflammatory response [11].

Flavonoids are anti-inflammatory compounds with a broad-spectrum antiviral activity against pathogens such as HIV-1, herpes simplex 1 and 2, influenza, dengue and yellow fever viruses [12,13,14,15]. NGN, a flavanone subclass of polyphenols [16] has demonstrated a promising antivirals effect, including inhibition of hepatitis C virus and limiting ZIKV replication [17,18,19]. NGN has also been shown to cross the blood–brain barrier [20] poises it as a promising candidate against ZIKV infection. However, its efficacy against congenital ZIKV infection remains under investigation.

This translational study aims to investigate whether inflammation in the placental microenvironment facilitates ZIKV infection and leads to adverse neonatal outcomes. The study will also assess the potential of modulating inflammation and antiviral responses using the flavonoid NGN to mitigate the harmful effects of infection and inflammation. Here, we showed that gestational ZIKV infection associated with CZS is characterized by severe histopathological alteration in the placenta, accompanied by the repression of immunological markers TNF, TLR4 and IL-10. Using an ex vivo system of placental explants and an in vivo model of ZIKV infection of pregnant C57BL/6 IFNAR knockout mice, we also showed that LPS-driven inflammation worsens the development of CZS. Importantly, NGN decreased viral replication and ameliorated developmental defects in newborns, supporting its potential as therapeutic drug against congenital ZIKV infection.

## 2. Methods

### 2.1. Human Subjects

Twenty-two ZIKV-infected pregnant women were enrolled in the Clinical Cohort Study of ZIKV-infected pregnant women and their infants at the maternal and child hospital Instituto Nacional de Saúde da Mulher, da Criança e do Adolescente Fernandes Figueira, Rio de Janeiro. Samples were collected in the period of 2015–2016, and congenital infection by ZIKV was confirmed during pregnancy by PCR analysis of urine, blood, or placenta samples. The mothers were also tested and excluded for HIV, past infections with Dengue or Chikungunya viruses, and other congenital infections known to cause infant neurological damage (TORCH). Placentas from pre-term and post-term deliveries were not considered. Samples were sorted between CZS and N-CZS groups based on the observation of microcephaly in the newborn, considered as head-circumference z-score of less than −2, determined at the moment of birth. The N-CZS group consisted of non-microcephalic children with no other features compatible with CZS, ensuring optimal contrast in the comparisons. The demographic data is shown in Supplementary Table S1.

For the ex vivo experiments, parturient woman with vaginal delivery (n = 16) and negative serology for syphilis, HIV-1, hepatitis B and C and toxoplasmosis, over 18 years of age and with gestational age over 37 weeks, were recruited at the Maternity Hospital of the University of São Paulo, Brazil. These control groups were used in the placenta explant cultures.

### 2.2. Experimental Design

No tools for sample size estimation were used for this study. The acquisition of samples was limited by the availability of cases (entries in the hospital facilities) during the period for this study execution. No randomization or blinding strategies were applied.

### 2.3. Ethics Statement

The human studies were conducted in accordance with the Declaration of Helsinki and approved by the Ethics Committee from the School of Public Health, University of São Paulo (approval number 59787216.2.0000.5421) and Instituto Nacional de Saúde da Mulher, da Criança e do Adolescente Fernandes Figueira, Fiocruz (approval number IRB/CAAE: 52675616.0.000.5269). Animal experiments were approved by the Ethics Committee of the Institute of Biomedical Sciences, University of Sao Paulo (approval number 05/2016).

### 2.4. Morphological Analysis of the Placenta

Histological sections of 4 µm thickness were taken from biopsies of paraffin-embedded placentas on silanized slides (Sigma Chemical Co., St. Louis, MO, USA). The histological sections were deparaffinized and stained with hematoxylin–eosin for microscopical analysis. The histopathological parameters of placental tissues from ZIKV-infected mothers who delivered babies with or without CZS were evaluated by a pathologist in a blinded manner.

### 2.5. Immunohistochemistry

All tissue specimens were formalin-fixed and embedded in paraffin, and 4 µm thick sections were obtained on silanized glass slides. The slides were dewaxed in xylene and hydrated through a graded series of ethanol washes. Endogenous peroxidase was blocked with 3% hydrogen peroxide. Antigen retrieval was performed in a water bath at 95 °C for 20 min, followed by washing in running water and distilled water for 5 min each. For TNF-*α* and TLR-4 analysis, the slides were incubated in Target Retrieval Solution, pH 6.0 (code S1699, Dako, Carpinteria, CA, USA). For IL-10 analysis, antigen retrieval was performed using Target Retrieval Solution, pH 9.0 (code S2367, Dako, Carpinteria, CA, USA).Then, they were incubated overnight at 4 °C in the presence of the primary antibody: polyclonal rabbit anti-TNF-*α* (1:300, ab9635, Abcam, Boston, MA, USA), monoclonal mouse anti-TLR4 (1:50, SC-293072, Santa Cruz, Heidelberg, Germany), or polyclonal goat anti-IL-10 (1:10, AF-217, R&D Systems, Minneapolis, MN, USA). The specific antigen–antibody reaction for TNF-*α* and TLR-4 was detected using the NovoLink Polymer Detection System Kit (code RE7280-CE, Leica Microsystems Inc., Newcastle Upon Tyne, UK), while IL-10 was detected with the Stain MAX PO Universal Immuno-peroxidase Polymer (Histofine 414161F, Nichirei Biosciences Inc., Tokyo, Japan), following the manufacturer’s instructions. The reactions were visualized using the 3,3′-diaminobenzidine-tetrahydrochloride (DAB) chromogen (Sigma, St. Louis, MI, USA) and counterstained with Carazzi hematoxylin. All reactions were accompanied by both positive (placenta and tonsil tissue) and negative controls. For the negative control of nonspecific binding, the primary antibodies were replaced with normal serum, under the same experimental conditions. For each protein analysis, we used five random regions on the slides for the placental villus and three regions for the decidua. The Image-Pro plus version 4.5.0.29 software was used for quantification, by calculating the positive area or optical density intensity (ODI) by the total area or length of the basement membrane for the placental decidua. Quantification was normalized using a 10× objective for decidual regions and 20× objective for placental villi.

### 2.6. Prediction of the Protein–Protein Interactions

The network was constructed using the SIGnaling Network Open Resource (Signor App v1.2) tool in Cytoscape 3.10.3.

### 2.7. Placental Explant Cultures

Placental cotyledons were extracted from randomly selected regions in DMEM/F12 culture medium (Gibco, Carlsbad, CA, USA). After separation into maternal (decidua) and fetal (villus) surfaces, the villus explants were dissected while preserving the syncytiotrophoblast structure. Approximately 100 mg of tissue was cultured in 24-well plates (Corning Costar, Tewksbury, MA, USA) containing DMEM/F12, 10% fetal bovine serum (SFB Gibco, CA, USA), 10 mg/mL gentamicin, 100 mg/mL penicillin/streptomycin, 1 mg/mL amphotericin B, 520 µg/mL sodium lactate and 56 µg/mL sodium pyruvate at 37 °C with 5% CO_2_ for 24 h. After, cells were washed twice and stimulated with lipopolysaccharide (LPS from *Salmonella* minnesota, Sigma-Aldrich, St. Louis, MO, USA) at three concentrations (0.1; 1 and 10 µg/mL) for 3 h at 37 °C with 5% CO_2_. Other experiments were supplemented with NGN (Sigma-Aldrich, St. Louis, MO, USA) at 120 mM plus LPS (1 μg/mL). The explants were washed twice and ZIKV (10^7^ or 10^8^ plaque-forming units [PFU]/mg of tissue) was added for 2 h and the wells were washed for removal of free/unbound viral particles. We used ZIKV strain BE H 815744, which is similar to the French Polynesian strain (Paraíba 2015) donated by Dr Clarisse Martins Machado (Virology Laboratory, IMT-USP). Cells were incubated for an additional 24 h in DMEM/F12 culture medium with 10% SFB at 37 °C with 5% CO_2_. Supernatants were then harvested for the following analyses.

### 2.8. RT-qPCR

The viral load in the culture supernatants was quantified using the RT-qPCR/Taqman technique. First, viral RNA was extracted from the supernatants using the QIAamp Viral RNA Kit (Qiagen, Maryland, USA) according to the manufacturer’s instructions. The TaqMan Fast Virus 1-Step Master Mix reagent (Thermo Fisher Scientific, Waltham, MA, USA) was used for the RT-PCR reactions, which were performed using the 7500 Real-Time PCR System (Applied Biosystem, Waltham, MA, USA). A previously published primer set was used to detect ZIKV RNA [21]. The primers and probes used in the reactions were: Forward 5′ CCGCTGCCCAACACAAG 3′; Reverse 5′ CCACTAACGTTCTTTGCAGACAT 3′ and probe FAM- AGCCTACCTTGACAAGCAGTCAGACACTCAA-MGB.

### 2.9. LPS Treatment, Infection and NGN Treatment of Pregnant Mice

Pregnant C57BL/6 IFNAR KO mice, 6–8 weeks of age, on day 12 of gestation were submitted for intravaginal injection with 2 µg of LPS. On the following day (day 13 of pregnancy), animals were challenged with 10^8^ PFUs of ZIKV by intravenous route. NGN or saline was administered via gavage (4 mg/kg) during a week, from days 13 to 19 of gestation. At the end of the pregnancy, a cesarean section was performed, and the fetuses were examined for signs of malformation, such as weight, crown-rump, biparietal diameter, skull height and skull length as described by Cugola et al., 2016 [22]. All measurements were performed in a blinded manner for the NGN and saline treatment groups.

### 2.10. Statistical Analyses

Statistical analyses were performed using GraphPad Prism software (version 9.0; GraphPad Software, San Diego, CA, USA). The comparison of two groups was performed with the non-parametric Mann–Whitney test and the comparison of three groups used the Kruskal–Wallis test with Dunn’s post-test. Fisher’s exact tests was used to compare proportions of categorical events between CZS and N-CZS participants. A *p*-value of 0.05 or less was considered significant.

## 3. Results

### 3.1. Histopathological Profile of the Placentas from ZIKV-Infected Mothers

Placenta samples harvested for this study were separated between those with or without CZS. In most of the cases linked to CZS, the infection occurred in the first trimester of pregnancy (72.7%), which was associated with the worst prognosis, while 27.3%, although asymptomatic, also developed CZS (Supplementary Table S1). In contrast, in the N-CZS group, 45.4% were infected in the second trimester and 45.4% in the third trimester.

**Figure 1:**
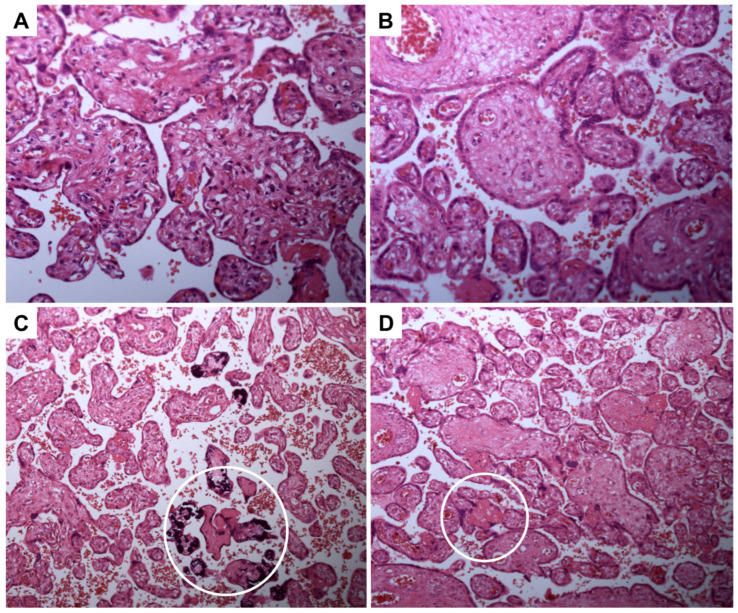
Placenta histopathology in ZIKV infection. Placentas from ZIKV-infected mothers were stained with H&E and analyzed by optical microscopy. (**A**,**B**) Presenting hyperplasia of Hoffbauer cells in villous CZS, 20×. (**C**) Circle showing evidence of calcification and hyalinization in N-CZS, 10×; (**D**) circle showing hyalinization in CZS, 10×.

The histopathological data of the specimens, shown in Table 1 and Figure 1A–D, indicated placental alterations regardless of CZS development. However, the CZS group displayed increased villitis (*p* = 0.0351), Hofbauer cell hyperplasia (*p* = 0.0351; intense versus moderate/mild levels), and decidual inflammation (*p* = 0.0124) compared with the N-CZS samples.

### 3.2. Altered IL-10 Expression in the Placentas from CZS Samples

Pre-existing placental inflammation or inflammation induced by a viral infection can adversely affect the pregnancy outcome. Therefore, we examined the expression of the pro-inflammatory factors TLR4 and TNF-α, and the anti-inflammatory cytokine IL-10 in placental tissue from ZIKV-infected mothers by immunohistochemistry, separating the maternal (decidua) and fetal (villus) sides of the tissue.

**Figure 2:**
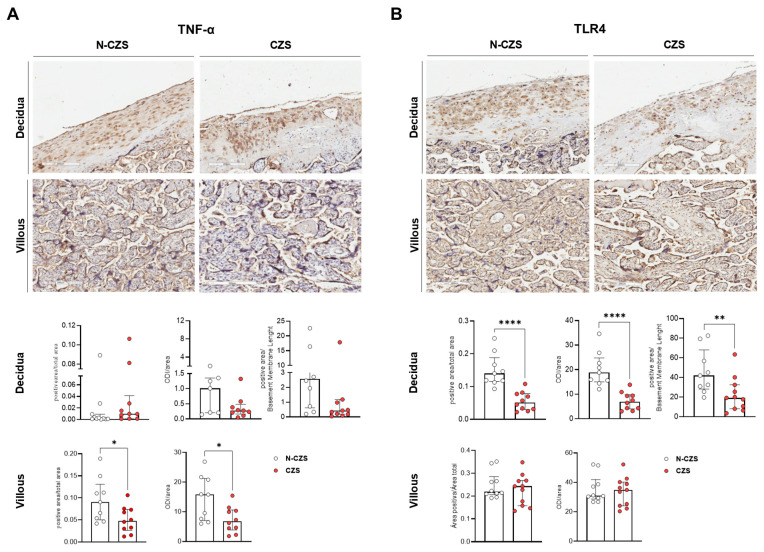
CZS placentas presented decreased expression of TNF in the villous and decreased TLR4 in the decidua. Placentas from ZIKV-infected mothers were divided into N-CZS, (white circles; n = 10) and CZS (red circles; n = 8) groups. Placental cotyledon fragments were isolated and sectioned into fetal and deciduous parts. (**A**) TNF and (**B**) TLR4 protein expression was analyzed in the decidua and the placental chorionic villi by immunohistochemistry, representing an average of three distinct regions of each slide for the decidua (10×) and five for the villi (20×). The bars represent the median and interquartile values for the positive area/total area, the positive ODI/total area for the decidua and villus, and the positive area/basement membrane length for the decidua. Mann–Whitney test: * *p* ≤ 0.05, ** *p* ≤ 0.01, **** *p* ≤ 0.0001.

Unexpectedly, while there were no differences on the decidual side, TNF-α was reduced on the villus side of the CZS group compared to the N-CZS one (Figure 2A). In contrast, the expression of TLR4 on the decidual side was reduced in placentas of CZS fetuses, but no changes were detected in the villous side (Figure 2B).

**Figure 3:**
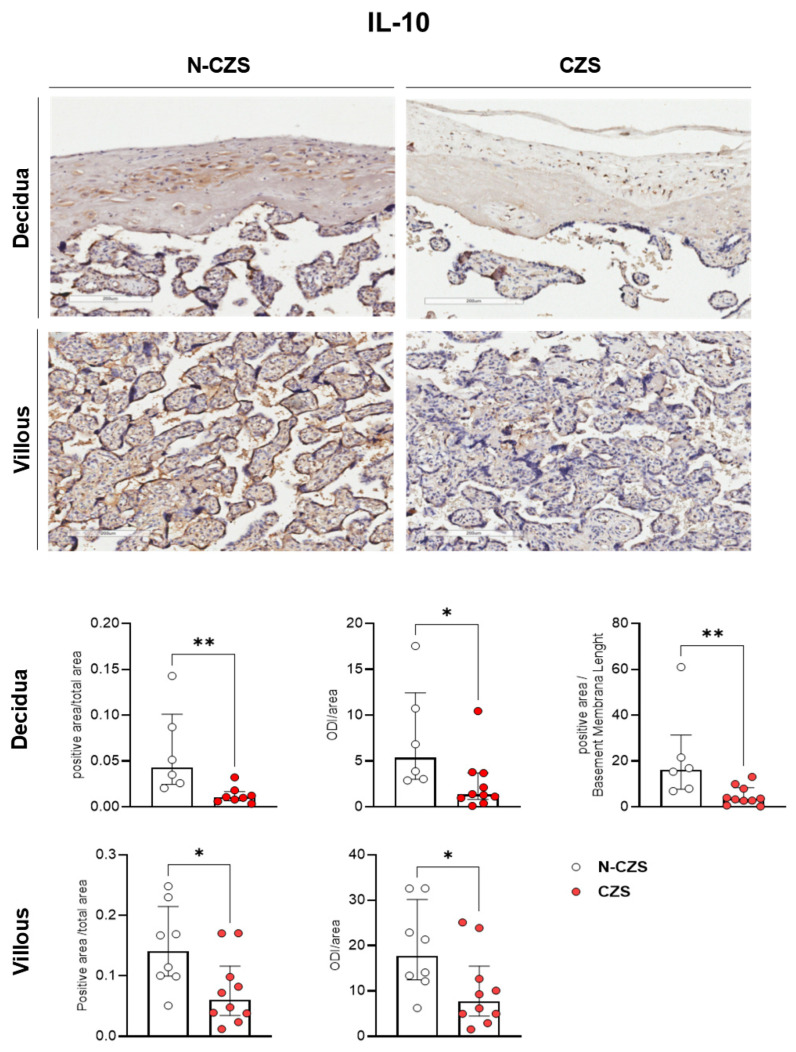
Down-regulation of IL10 expression in the placenta of ZIKV-infected mothers. Placentas from ZIKV-infected mothers were divided into N-CZS (white circles; n = 8) and CZS (red circles; n = 10) groups. Placental cotyledon fragments were isolated from the fetal and decidua parts. IL-10 protein expression was analyzed in the decidua and the placental chorionic villi by immunohistochemistry, representing an average of three distinct regions of each slide for the decidua (10×) and five for the villi (20×). The bars represent the median and interquartile values for the positive area/total area, the positive ODI/total area for the decidua and villus, and the positive area/basement membrane length for the decidua. Mann–Whitney test: * *p* ≤ 0.05, ** *p* ≤ 0.01.

Intriguingly, the expression of IL-10, both in the decidua and villous layers, was also significantly diminished in the CZS samples (Figure 3). Besides its immunosuppressive function, IL-10 is also pivotal for a successful pregnancy [23]. Thus, the reduction we observed could be evidence of the dysfunctional regulation of the placental milieu in CZS.

**Figure 4:**
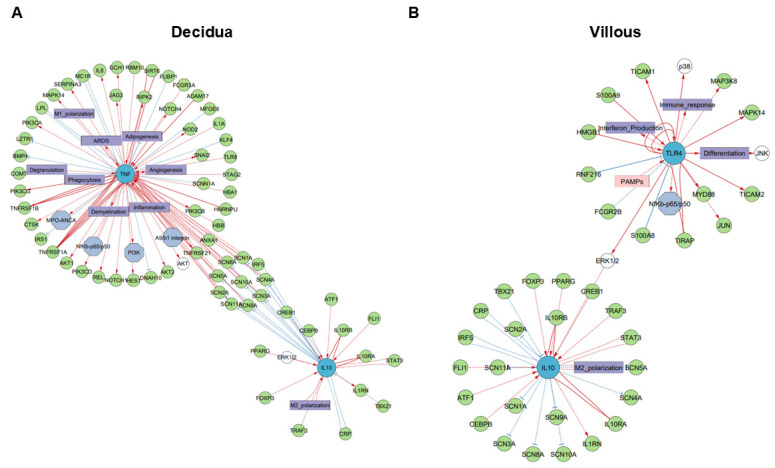
Prediction of protein–protein interactions involving the downregulated proteins TNF-α, IL-10, and TLR4 and their associated interactors. (**A**) TNF-α and IL-10, downregulated on the decidual tissue of the placenta, along with their interacting proteins; (**B**) TLR4 and IL-10, detected in the villous tissue, and their respective protein interactors. Blue nodes: downregulated proteins identified in the study, green nodes: predicted interacting proteins, gray nodes: protein complexes, and white nodes: protein families interacting with the downregulated proteins. Purple rectangles: pathways that either regulate or are regulated by these proteins. Red arrows: indicate induction, blue arrows: indicate suppression.

To better understand the potential biological impact of the observed alterations in the placenta, we performed an exploratory in silico simulation of potential protein–protein interactions of the analyzed proteins and their targets (Figure 4A for decidua and Figure 4B for villous). This analysis aimed to identify previously described interaction networks associated with the altered proteins detected.

As expected, TNF was linked to inflammatory pathways; TLR4 to NF-κB signaling molecules, and IL-10 to STAT3 signaling-related proteins. Interestingly, both TNF and IL-10 demonstrated complementary interactions with members of the sodium channels (SCN) family. These channels are critical for maintaining placenta homeostasis [24] and their dysregulation has been implicated in pathological conditions such as preeclampsia [25], particularly due to their role in supporting trophoblast function. In addition to classical immune effector functions, the alterations in TNF, IL-10 and TLR4 related to CZS could also compromise the stability of the syncytiotrophoblast layer, favoring the pathological phenotype. However, it is important to note that these observations are based on predicted interactions, and further experimental validation is required.

Overall, our results suggest that CZS may contribute to alterations in the placenta phenotype, with enhanced anatomopathological changes and dysbalanced immune status.

### 3.3. NGN Modulates ZIKV Replication in Placental Explants Exposed to LPS

**Figure 5:**
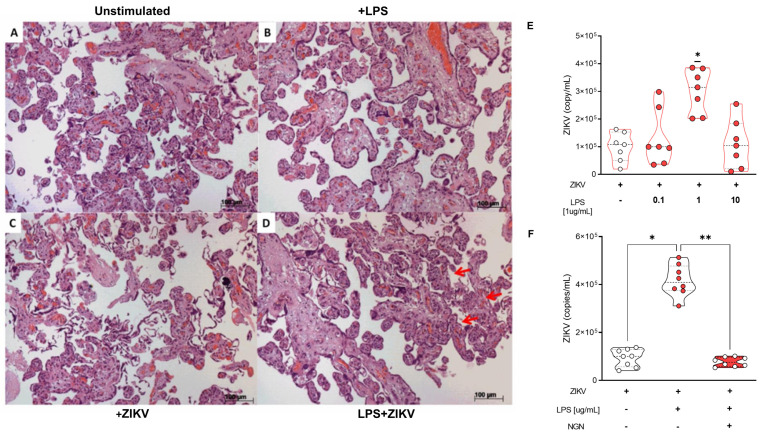
LPS-induced inflammation of placental explants leads to increase in vitro ZIKV replication. Placental villus explants (n = 4) were maintained in culture medium for 24 h and stimulated with LPS (1 μg/mL) for 3 h. After washing, the explants were infected with ZIKV (10^8^ PFU/mg tissue) for 2 h at 37 °C, washed and cultured for 24 h. H&E staining, representative of 4 assays, at 10× magnification. (**A**) Placenta explant unstimulated (basal), (**B**) placenta explant stimulated with LPS, (**C**) placenta explant infected with ZIKV, (**D**) explant stimulated with LPS and infected with ZIKV. In this condition, stimulation by LPS followed by ZIKV infection induces focal and diffuse edema and hyperplasia of Hofbauer cells (red arrowsred) in placental villus explants. (**E**) To verify LPS dose stimulation, placental explants were cultured with 0.1, 1 and 10 μg LPS/mL and infected with ZIKV. (**F**) To verify the NGN effects: placental explants were stimulated with LPS (1 μg/mL) plus NGN (120 mM) and infected with ZIKV. Data shows median and interquartile range. Kruskal–Wallis and Dunn’s post-test: * *p* ≤ 0.05, ** *p* ≤ 0.01.

To explore potential interventions to rescue the compromised phenotype of CZS placentas, and to mimic the effects of in vivo placenta, we employed a system using trophoblast explants. First, we assessed whether our system could reproduce the pathological features we observed in our patients. Placental explant were stimulated with LPS for 3 h and further exposed to ZIKV for 2 h showed focal and diffuse edema and displayed hyperplasia of Hofbauer cells (red arrows) (Figure 5A–D), as we observed in vivo. Next, explants were stimulated with 0.1, 1.0 or 10 μg/mL, and infected. Intriguingly, LPS at 1 μg/mL increased ZIKV replication compared to unstimulated conditions (Figure 5E).

We hypothesized that correction of the dysfunctional inflammation could counterbalance the infection enhancement. We then investigated whether the anti-inflammatory action of NGN would reverse the results. Indeed, Figure 5F shows that the addition of NGN to LPS-stimulated at 1 μg/mL explants was able to decrease the viral replication. Overall, our data shows that exacerbated placental inflammation at the optimal dose of 1 μg/mL boosts ZIKV replication which is blunted by NGN treatment.

### 3.4. Translational Effect of NGN to Inflammatory Response of Mice Infected with ZIKV

**Figure 6:**
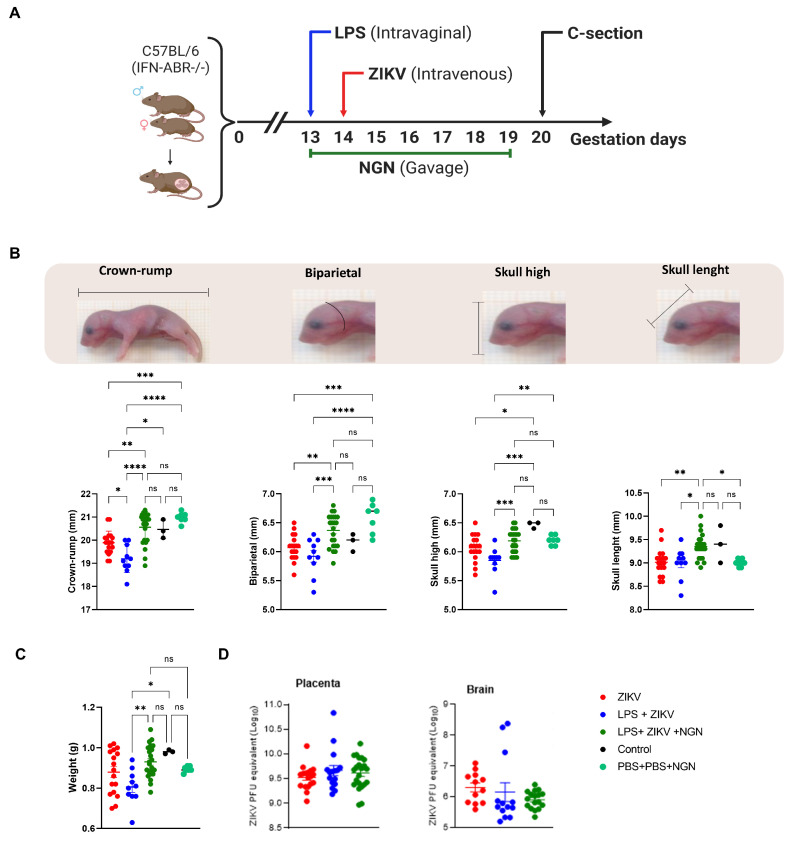
NGN decreased brain defects of ZIKV infection enhanced by LPS-induced inflammation. (**A**) Scheme for maternal LPS administration, ZIKV infection and NGN treatment. Pregnant C57BL/6 IFNRA KO mice were intravaginal injected with LPS on day 12 of pregnancy the following day were challenged with 10^8^ ZIKV PFUs by intravenous route. NGN was administered via gavage (0.08 mg/dose) on days 13 to 19 of gestation. At the end of the period, fetuses were collected by cesarean procedure (**B**) fetuses were examined for signs of malformation, such as crown-rump, biparietal diameter, skull height and skull length. ZIKV-infected group (n = 18), ZIKV + LPS (n = 11), ZIKV + LPS + NGN (n = 23), and control uninfected unstimulated (n = 3) groups received only NGN PBS + PBS + NGN (n = 7). (**C**) weight and (**D**) viral load assessed in placenta (n = 16–23) and brain (n = 12–17) by RT-qPCR/Taqman. Data are shown as mean ± SD. Kruskal–Wallis and Dunn’s post-test: * *p* ≤ 0.05, ** *p* ≤ 0.01, *** *p* ≤ 0.001 and **** *p* ≤ 0.0001, ns, not significant.

Finally, we aimed to translate the previous findings to an in vivo system. Using an established model of ZIKV infection, we previously induced inflammatory response by intravaginally injecting LPS to pregnant C57BL/6 IFNRA1 -/- KO mice on day 12 of pregnancy and infecting them with ZIKV the following day (Figure 6A). As verified in the human placenta explant, the NGN effect was also analyzed in the pregnant mice, the NGN effect, administered NGN by gavage (4 mg/kg/day) during days 13–19 of gestation.

In this murine model, body and brain measurements in the offspring (Figure 6B) showed evidence of fetal microcephaly upon ZIKV challenge. Administration of LPS significantly decreased crown-rump measurements, indicating that LPS did aggravate the ZIKV-driven fetal microcephaly. Besides a small number of uninfected and unstimulated control mice, resulted in an increased crown-rump length and skull height compared with LPS and ZIKV infection. The other control group was those that received only NGN (PBS + PBS + NGN), this group is similar to control unstimulated and uninfected mice, as well as the NGN treated and infected mice. This finding shows that the infected mice under NGN treatment was similarly to the control—uninfected mice.

The therapeutic effect of maternal NGN treatment was remarkable. The flavonoid administration restored all evaluated fetal morphological parameters such as crown, biparietal diameter, skull length or skull height and weight when compared to both ZIKV-infected mice and mainly in the group exposed prior LPS-induced inflammation. The effect of NGN plus LPS on ZIKV infection increased crown, biparietal diameter, skull height and length. In addition, NGN treatment increased fetal weight when compared with LPS and ZIKV infection (Figure 6C). Unexpectedly, viral loads in the placentals and brains tissues were not affected by LPS or NGN treatment (Figure 6D).

In summary, our results showed that LPS-induced inflammation enhances ZIKV-driven fetal outcomes that can be improved by NGN treatment.

## 4. Discussion

The intense transmission of ZIKV during the 2015/2016 epidemics was followed by a sustained low transmission rate. A low ZIKV seroprevalence has been noted in northeastern Brazil [26], indicating that a significant proportion of the population is still susceptible to ZIKV infection. Therefore, many questions must still be researched regarding the pathophysiological basis of CZS. Our results showed that CZS is associated with more severe placental alterations and a dysfunctional immune response at the maternal–fetal interface. We also showed that the pharmacological intervention with the flavonoid NGN has the potential to protect the developing fetus from the ZIKV-induced anomalies.

In the cohort of our study, anatomopathological features such as Hofbauer cell hyperplasia, villitis and decidual inflammation were more intensively observed in the CZS group. In agreement with our findings, Hofbauer cell hyperplasia has been shown previously to occur in second and third trimester placentas in cases of intrauterine ZIKV infection [27,28], also being associated with inflammatory damage to the tissue [8,29].

The decreased expression of IL-10 in CZS placentas could be critical for the observed phenotypes since the cytokine is known to play a key role in facilitating successful pregnancies due to its immunosuppressive function [23]. A previous work with our cohort showed that the IFNGN1 rs2257167 CG/CC genotype is linked to high levels of type I IFN, but low type III IFN, in the placenta, which could favor CZS development [30]. Our protein–protein interaction prediction indicates that TBX21 (T-Box Transcription Factor 21), a key transcriptional activator of Th1 cell differentiation and IFN responses, could inhibit IL-10 expression. Thus, IL-10 repression in placenta could be a consequence of the enhanced type I IFN response. Moreover, the repression of IL-10 could represent a viral escape strategy that hijacks the innate immune system and promotes chronic placental infection. It is known that the human cytomegalovirus encoded cytokine IL-10 can modulate the immune response [31,32].

The repressed IL-10 expression can also be interpreted as a tendency towards enhanced placental inflammation in CZS. In accordance with this hypothesis, this maternal inflammation could provide a microenvironmental to CZS, verified by our finding in vitro model of placenta explants that stimulation with LPS prior to ZIKV infection favored viral replication and reproduced the anatomical alterations (diffuse edema and hyperplasia of resident macrophages) observed in the native tissue. One possible reason for the increase in ZIKV replication following LPS stimulation could be due to metabolic changes. LPS stimulation may increase the rate of glycolysis, which is favorable to ZIKV infection [33]. ZIKV infection is known to evoke a glycolytic response, as evidenced by an elevated extracellular acidification rate and increased expression of key glycolytic genes (GLUT1, HK2, TPI and MCT4) [34].

In fact, an increased expression of glucose transporter type 1 (*Slc2a1*/Glut1) and decreased levels of glucose-6-phosphate in female placentae have been shown in ZIKV-infected pregnant mice. Furthermore, fetal–placental growth was impaired in male fetuses. Therefore, sex-specific effects of maternal ZIKV exposure on fetal–placental growth, placental nutrient transporter expression, intraplacental nutrient distribution, placental HBP function, and O-GlcNAcylation [35].

Nevertheless, it should be pointed out that we verified a reduced expression of TNF-α and TLR4 in the placentas of CZS fetuses, in contrast to our initial expectations. However, since we worked with term placentas, we can hypothesize that it could be a consequence of the prolonged inflammation those mothers experienced during their pregnancies. We do not have clinical information regarding urinary tract infections (UTIs) during pregnancy in this patient cohort, which may represent a limitation of our study. Studies on UTIs during pregnancy in northeastern Brazil have shown that *Escherichia coli* is the most common uropathogen, accounting for approximately 87% of positive urine cultures [36].

We must consider that bacterial urinary infection or maternal periodontal disease are common events during pregnancy, predisposing the mothers to LPS exposure; it is known that persistent LPS stimulation can reduce TLR4 expression [37]. As predicted by our protein–protein interaction map, factors such as RNF216 (Ring Finger Protein 216) and S100A8 can control TLR4 expression. RNF216 promotes the degradation of TRAF3, TLR4 and TLR9 [38], which down-regulates NF-kB and IRF3 activation [39].

Finally, we wanted to consider whether the intervention in the inflammatory response could alter the outcome of ZIKV infection. We chose the mouse strain C57BL/6 IFNRA1 -/- KO mice due to the absence of the type I IFN receptor. This IFN deficiency may lead to more severe or faster phenotypes, affecting our understanding of pathogenesis. Additionally, IFN regulates inflammation [40], so its absence could lead to an increased inflammatory response. However, NGN was able to mitigate the inflammatory response. Given the broad anti-inflammatory effects of NGN, we attempted NGN treatment. Furthermore, this approach had not previously been observed in an in vivo ZIKV model. However, several studies have investigated its broad anti-inflammatory and antioxidant effects, particularly using in vitro models.

Indeed, the administration of the anti-inflammatory flavonoid NGN could mitigate the increase in ZIKV replication and the pathological features of the infection in vitro [41]. Mechanistically, NGN can activate the PI3K-Akt-mTOR pathway, inhibiting autophagy and promoting Nrf2 expression, which decreases the cellular oxidative stress [42]. The strong antioxidant effect of NGN can, therefore, provide neuroprotection-like observations of abrogated ischemic brain injury and suppressed NF-kB-mediated neuroinflammation in a model of prophylactic NGN administration [43]. In addition, NGN could also exert a direct virucidal effect. Molecular docking analysis suggested a possible interaction between NGN and the protease domain of the NS2B-NS3 protein of ZIKV, hinting at a function as a non-competitive inhibitor of the enzyme that interferes with the late phase of the viral life cycle [41].

Bioactive constituents like naringenin, mangiferin, α-mangostin, geraniin, punicalagin, and lectins of edible fruits exhibit antiviral effects by inhibiting viral replication against IFV, DENV, polio, CHIKV, ZIKV, HIV, HSV, HBV, HCV, and SARS-CoV [44]. Moreover, NGN—and also quercetin and pinocembrin—emerge as promising ZIKV E protein inhibitors with robust target engagement and favorable drug-like profiles [45]. Despite extensive analyses of the in vitro effects of NGN, there are few reports on experimental models of viral infections in vivo, particularly with ZIKV.

Besides being anti-infective, NGN’s importance also lies in its anti-inflammatory properties [16]. NGN shows—in a dose-dependent manner—inhibition of the replication of pseudorabies virus in the brain, lungs, and kidneys of the mice and alleviated the pathological changes, hereby inhibiting NF-κB pathway activation. This intervention subsequently attenuated the expression levels of pro-inflammatory mediators [46].

Although the therapeutic use of NGN may be limited due to its poor absorption, low bioavailability, and inability to cross physiological barriers, our results in vivo showed that administration by gavage was protective during pregnancy. Indeed, NGN has been used during pregnancy in other disease conditions such as gestational diabetes [47] and gestational hypertension [48], where NGN alleviated the clinical symptoms.

Evidence that in vitro LPS increases ZIKV replication may be correlated with ex vivo urinary tract infection. The high prevalence of Gram-negative bacterial infections in the poorest region during pregnancy could be a contributing factor to the high prevalence of ZIKV in north-eastern Brazil.

The results demonstrated the crucial role of NGN in protecting the fetus’s cranium during development. All parameters, including crown-rump length, biparietal diameter, skull height, and skull length, showed improvement in relation to infection and inflammation associated with infection. Treatment with NGN produced results similar to those observed in uninfected control groups or groups treated only with NGN. Our findings suggest that NGN reduces neurotoxicity in brain tissue and improves weight, probably due to its anti-inflammatory properties. This is relevant because congenital ZIKV infection is characterized by intrauterine growth restriction, including microcephalic features, in mice [22]. The neuroprotective effects of NGN, including its anti-amyloidogenic, antidepressant and neurotrophic properties [49], may also offer protection against various neurological diseases. However, NGN did not alter the viral load in the brain or placenta. It is important to note a limitation of this study: we should have assessed viral infectivity using plaque-forming units (PFUs), as this represents a functional measure of viral infection. Viral load quantification may also detect inactive or defective viral particles. Another limitation of the study is the lack of statistical power due to the small number of ZIKV-infected placentas, mainly because of the rarity of samples associated with the development of CZS.

Our findings from the placentas of mothers who developed CZS were consistent with the ex vivo analysis of trophoblastic explants from full-term placentas. Histopathological findings revealed inflammatory processes, Hofbauer cell hyperplasia, villitis, and decidual inflammation in the placentas of mothers who delivered infants with CZS. This finding was also obtained in infected placental explants. Inflammation with LPS in vitro and in the in vivo model of pregnant women favored an increase in the ZIKV viral load and microcephaly parameters in newborns, respectively. Findings of cytokine dysfunction in infected placentas and mothers, as well as in vitro and in vivo findings of ZIKV infection, support the analysis of drugs with a particularly anti-inflammatory antioxidant effect, such as NGN. Once again, the data obtained in vitro with placental explants were consistent with the attenuation of microcephaly findings observed in the CZS in vivo model in mice. Considering its remarkable anti-inflammatory effect, the action of NGN could be applicable to viral infections in general that occur during pregnancy.

Overall, our data demonstrated that CZS is related to an aberrant placental phenotype, probably due to an altered immune response. LPS-driven inflammation can favor ZIKV infection and its pathological consequences to the fetus, but it can also be targeted for pharmacological intervention, where NGN as a suitable candidate for anti-inflammatory during pregnancy.

## Figures and Tables

**Table 1:** Histopathological analysis of placentas from mothers with ZIKV infection.

Characteristic	N-CZS			CZS			Fisher’s Exact Test
	Score	n/N	%	Score	n/N	%	*p*-Value
Trophoblast necrosis	+	(2/11)	18.2	+	(0/11)	0	*p* > 0.05
	+ +	(5/11)	45.4	+ +	(9/11)	81.8	
	+ + +	(4/11)	36.4	+ + +	(2/11)	18.2	
Villitis	-	(11/11)	100	-	(6/11)	54.5	*p* = 0.0351
	+	(0/11)	0	+	(5/11)	45.4	
Intervillositis	-	(11/11)	100	-	(10/11)	90.9	*p* > 0.05
	+	(0/11)	0	+	(1/11)	9.1	
Intervillous thrombi	-	(2/11)	18.2	-	(1/11)	9.1	*p* > 0.05
	+	(3/11)	27.2	+	(2/11)	18.2	
	+ +	(4/11)	36.4	+ +	(8/11)	72.7	
	+ + +	(2/11)	18.2	+ + +	(0/11)	0	
Hofbauer cell hyperplasia	+	(1/11)	9.1	+	(0/11)	0	*p* = 0.0351
	+ +	(10/11)	90.9	+ +	(6/11)	54.5	
	+ + +	(0/11)	0	+ + +	(5/11)	45.4	
Fibrinoid necrosis of villi	-	(2/11)	18.2	-	(0/11)	0	*p* > 0.05
	+	(4/11)	36.4	+	(3/11)	27.2	
	+ +	(5/11)	45.4	+ +	(8/11)	72.7	
Villi calcification	-	(2/11)	18.2	-	(0/11)	0	*p* > 0.05
	+	(2/11)	18.2	+	(6/11)	54.5	
	+ +	(2/11)	18.2	+ +	(3/11)	27.2	
	+ + +	(5/11)	45.4	+ + +	(2/11)	18.2	
Fetal surface changes	-	(11/11)	100	-	(11/11)	100	
Syncytial knots	+ +	(7/11)	63.6	+ +	(3/11)	27.2	*p* > 0.05
	+ + +	(4/11)	36.4	+ + +	(8/11)	72.7	
Decidual inflammation	-	(11/11)	100	-	(5/11)	45.4	*p* = 0.0124
	+	(0/11)	0	+	(6/11)	54.5	

- non-present; + low level; + + moderate level; + + + intense level, samples of placental with no cephalic alterations (N-CZS, n = 11) and microcephaly/hydrocephalus (CZS, n = 11).

## Data Availability

The datasets generated and/or analyzed during the current study are not publicly available due to ethical and privacy restrictions related to the use of human placental samples and sensitive clinical data, but are available from the corresponding author on reasonable request.

## Supplementary Materials

The following supporting information can be downloaded at: https://www.mdpi.com/article/10.3390/v18060615/s1, Table S1: Demographic characteristics of mothers and newborns at birth.
