# Supplementary material for: Inflammation Exacerbates Congenital Zika Virus Infection and Naringenin Provides Protective Effects
Source: Viruses. 2026 May 28;18(6):615. doi: 10.3390/v18060615 (PMC13307879; doi:10.3390/v18060615)
Supplement: Supplementary file 1 [file viruses-18-00615-s001.zip › viruses-4288998-supplementary.pdf]

Supplementary Table S1. Demographic characteristics of mothers and newborns at birth.

Characteristics	ZIKV+ N-CZS		ZIKV+ CZS	
	n/N	%	n/N	%
**Mother**				
Types of parturition				
Cesarean section	(8/11)	72.7	(3/11)	27.3
Vaginal delivery	(0/11)	0	(5/11)	45.4
N/A	(3/11)	27.3	(3/11)	27.3
Gestational age (weeks)	37.9 ± 2.5	-	39.1 ± 1.8	-
Infection trimester				
1st	(1/11)	9.1	(8/11)	72.7
2nd	(5/11)	45.4	(0/11)	0
3rd	(5/11)	45.4	(0/11)	0
Asymptomatic	(0/11)	0	(3/11)	27.3
ZIKV-positive placenta PCR	(4/11)	36.4	(3/11)	27.3
Parity	1.4 ± 0.8	-	1.6 ± 0.7	-
Previous abortions	(1/11)	9.1	(0/11)	9.1
Smokers during pregnancy	(0/11)	0	(0/11)	0
Alcohol during pregnancy	(0/11)	0	(0/11)	0
**Newborn**				
Apgar Score				
1’	8.4 ± 1.2	-	7.9 ± 1.0	-
5’	7.7 ± 3.2	-	8.7 ± 0.5	-
Weight (g)	3096.4 ± 665.5	-	2826.9 ± 348.6	-
Micro/Hidrocephaly	(0/11)	0	(11/11)	100
Ocular abnormalities	(0/11)	0	(9/11)	81.8
